# Supplementary material for: Understanding Infection Progression under Strong Control Measures through Universal COVID‐19 Growth Signatures
Source: Glob Chall. 2021 Mar 1;5(5):2000101. doi: 10.1002/gch2.202000101 (PMC7995214; doi:10.1002/gch2.202000101)
Supplement: Supplementary file 1 — Supporting Information [file GCH2-5-2000101-s001.pdf]

© 2021 Wiley-VCH GmbH

Global Challenges
Open Access

Supporting Information

for *Global Challenges,* DOI: 10.1002/gch2.202000101

Understanding Infection Progression under Strong Control Measures through Universal COVID-19 Growth Signatures

Magdalena Djordjevic, Marko Djordjevic,* Bojana Ilic, Stefan Stojku, and Igor Salom*

For each Figure, the caption is equivalent to Fig. 1 in the manuscript.

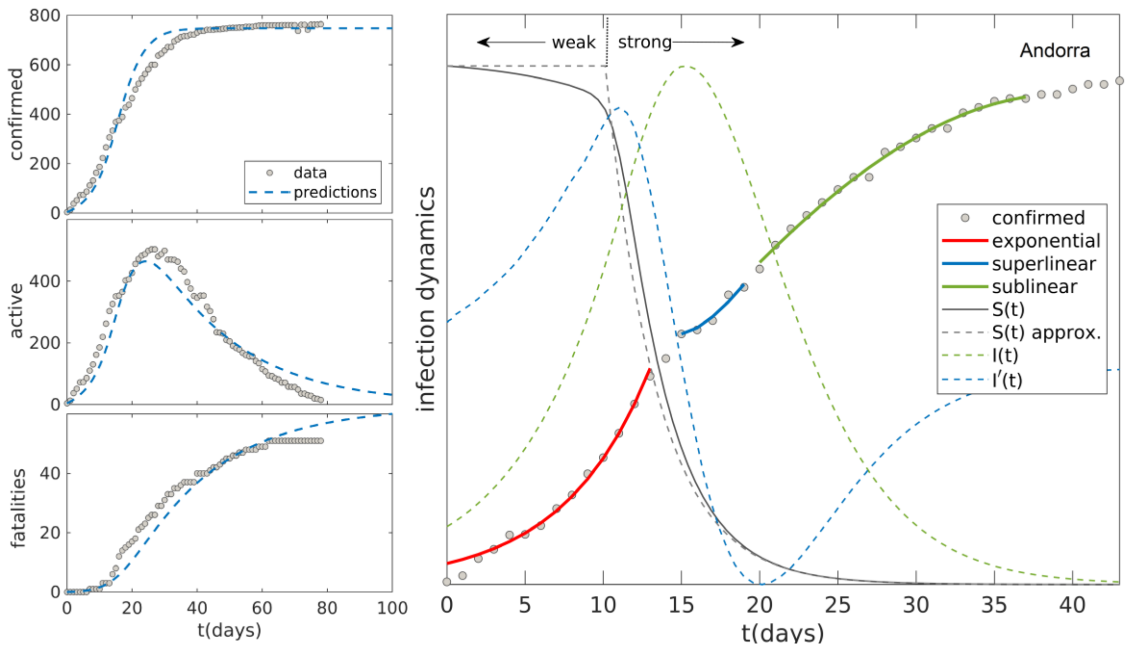

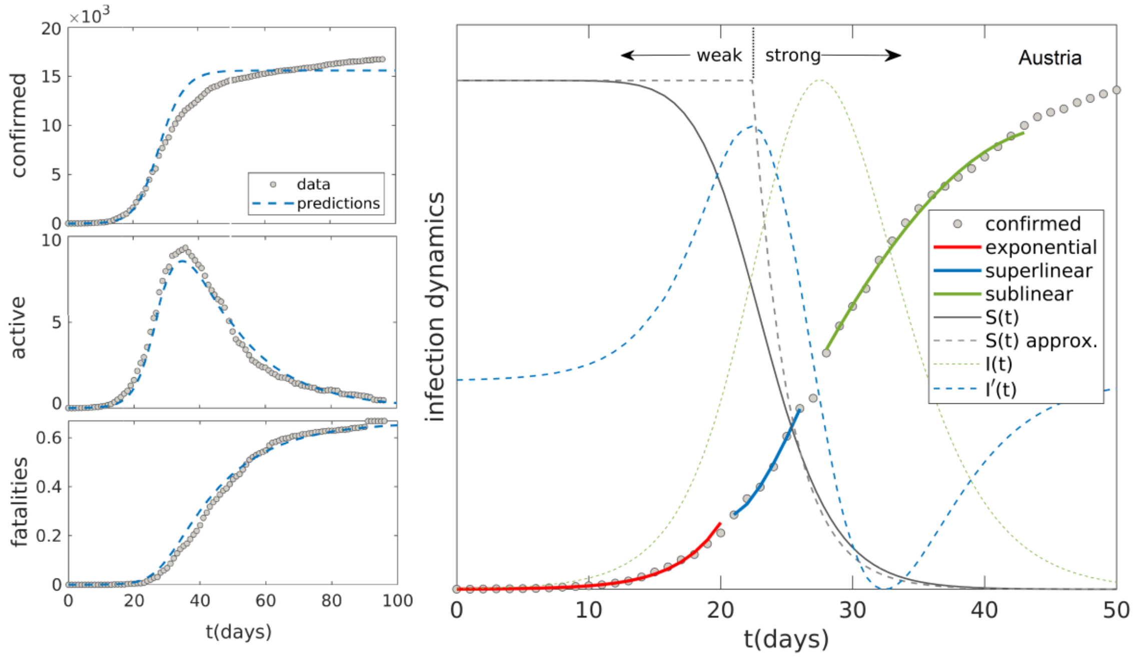

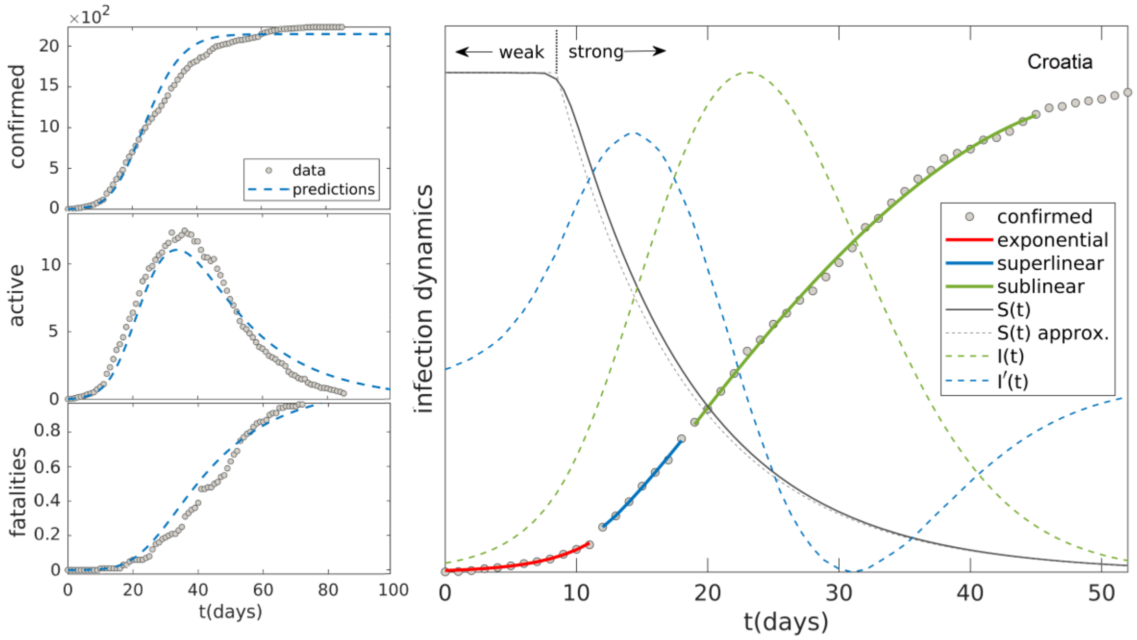

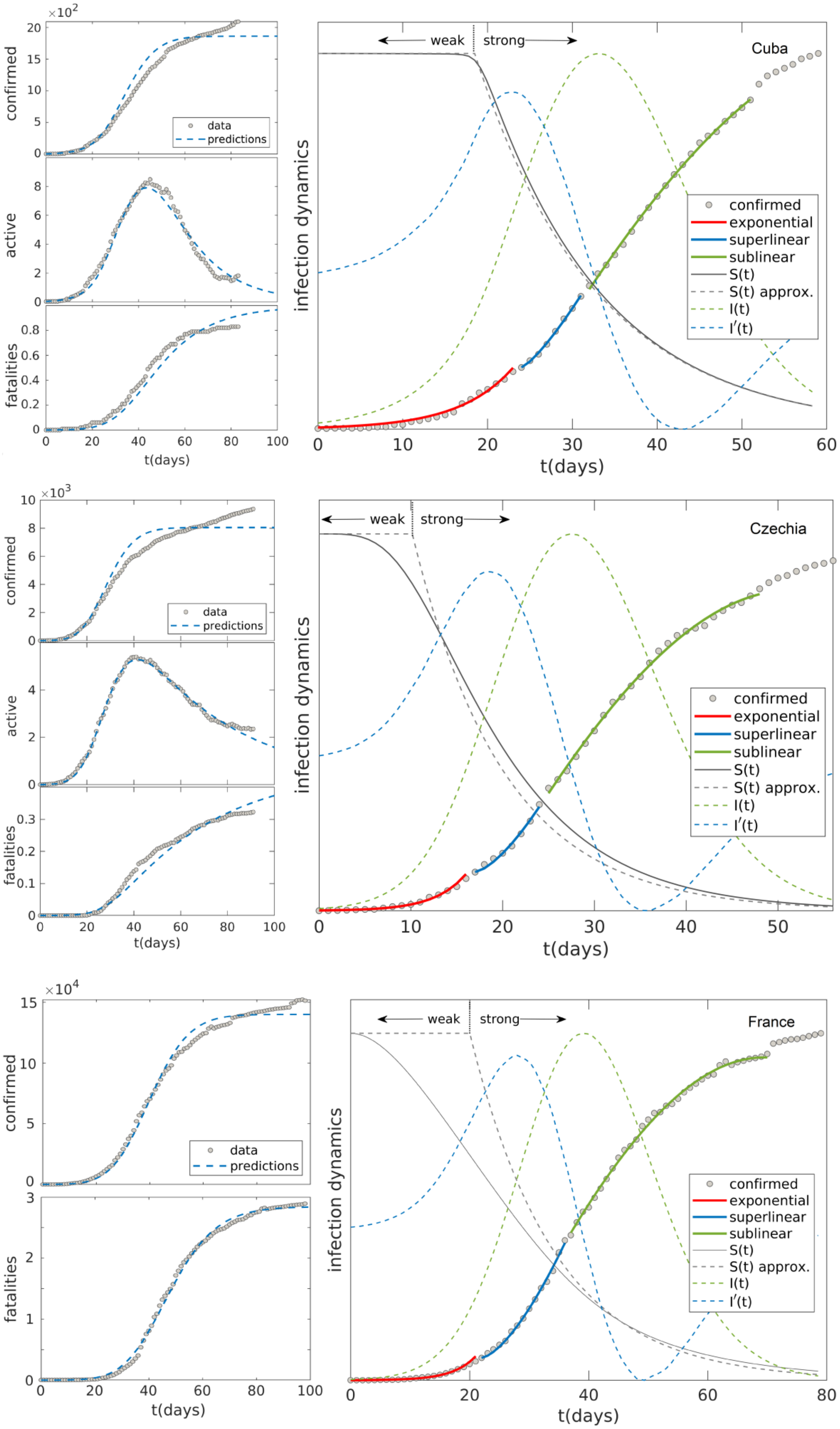

×10²
confirmed
active
fatalities
t(days)
data
predictions
infection dynamics
weak
strong
Cuba
confirmed
exponential
superlinear
sublinear
S(t)
S(t) approx.
I(t)
I′(t)
×10³
Czechia
×10⁴
France

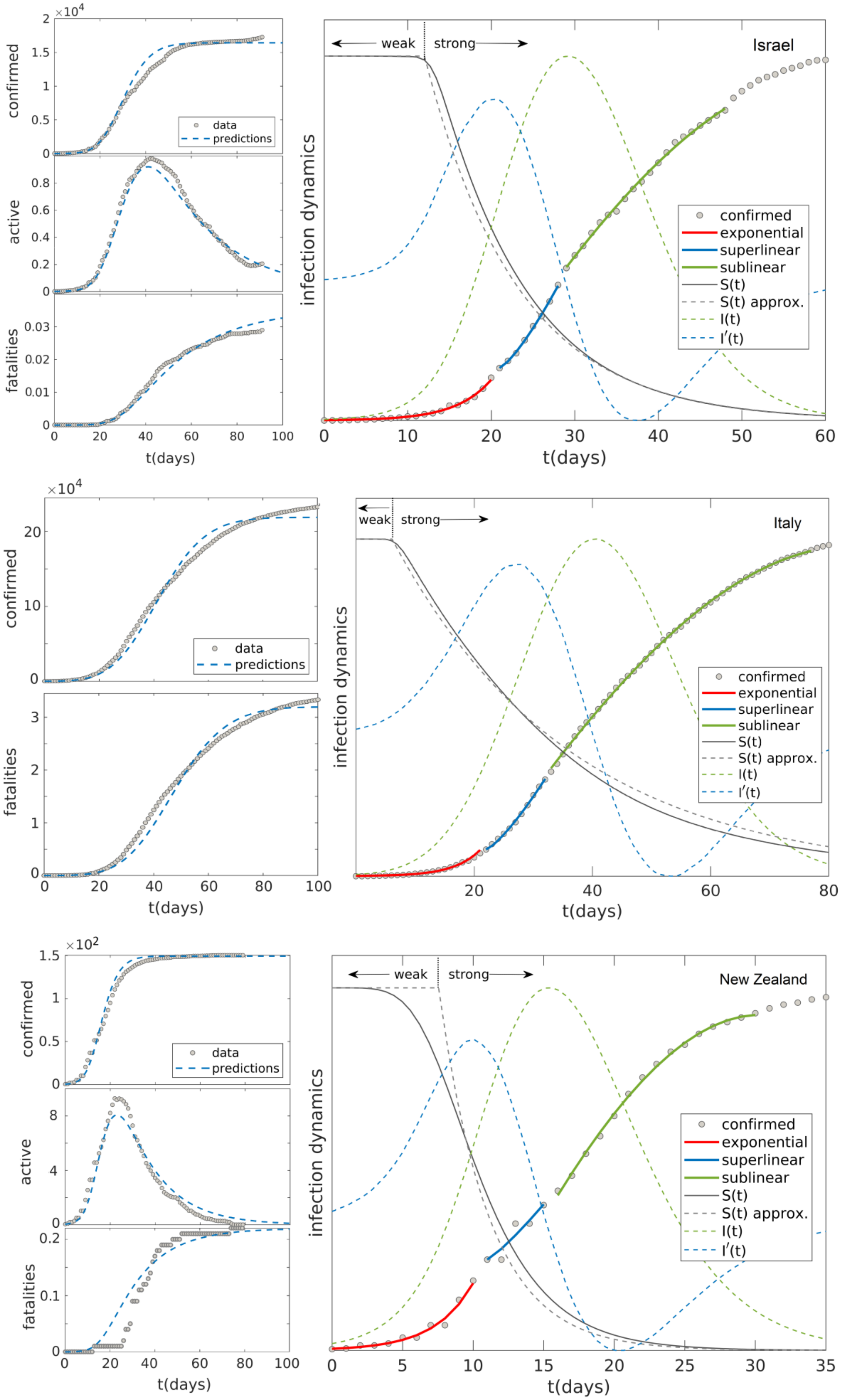

×10^4
confirmed
active
fatalities
data
predictions
t(days)
infection dynamics
weak
strong
Israel
confirmed
exponential
superlinear
sublinear
S(t)
S(t) approx.
I(t)
I′(t)
t(days)
×10^4
confirmed
fatalities
data
predictions
t(days)
infection dynamics
weak
strong
Italy
confirmed
exponential
superlinear
sublinear
S(t)
S(t) approx.
I(t)
I′(t)
t(days)
×10^2
confirmed
active
fatalities
data
predictions
t(days)
infection dynamics
weak
strong
New Zealand
confirmed
exponential
superlinear
sublinear
S(t)
S(t) approx.
I(t)
I′(t)
t(days)

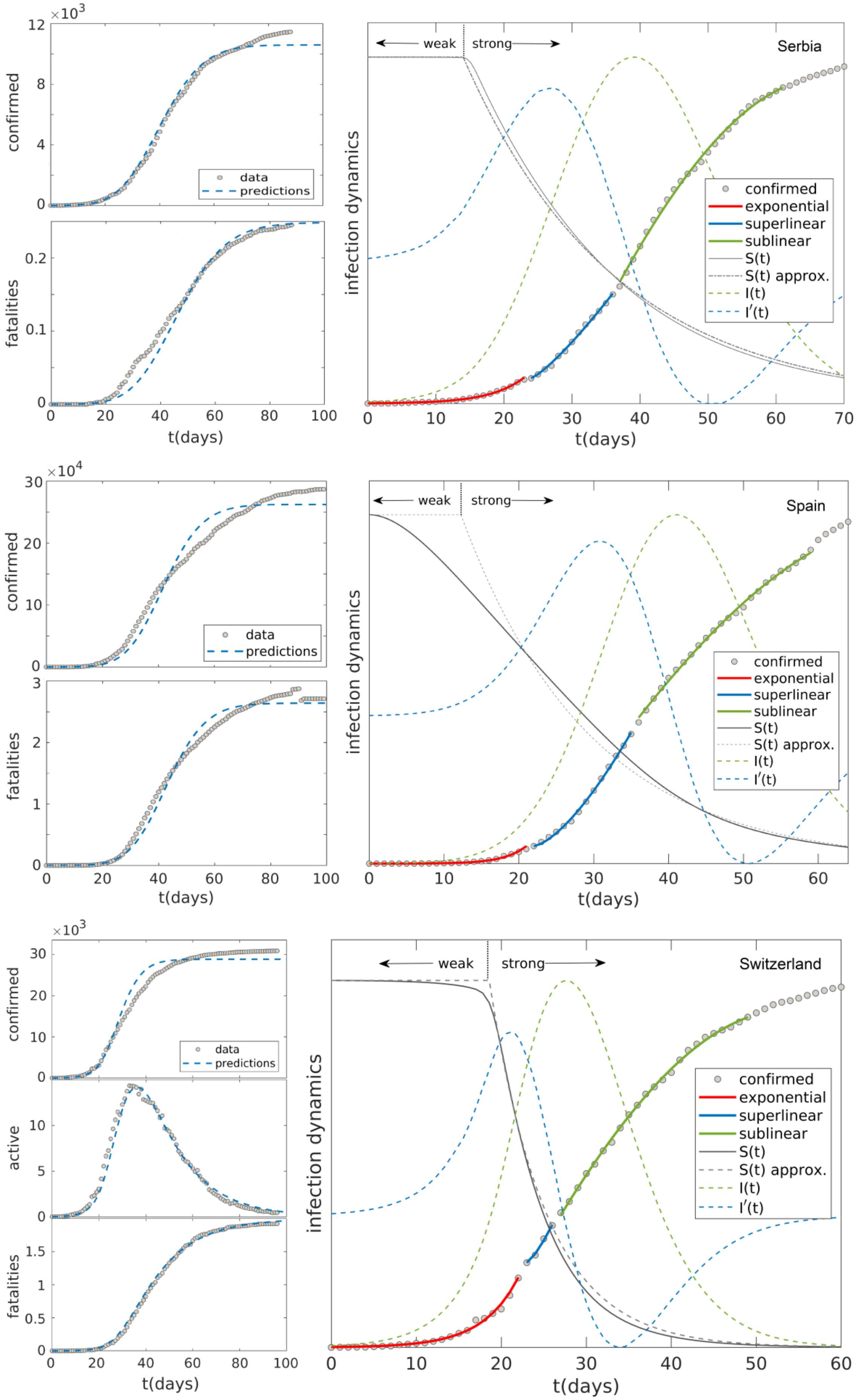
×10^3
confirmed
fatalities
data
predictions
t(days)
infection dynamics
weak
strong
Serbia
confirmed
exponential
superlinear
sublinear
S(t)
S(t) approx.
I(t)
I′(t)
×10^4
Spain
×10^3
active
Switzerland

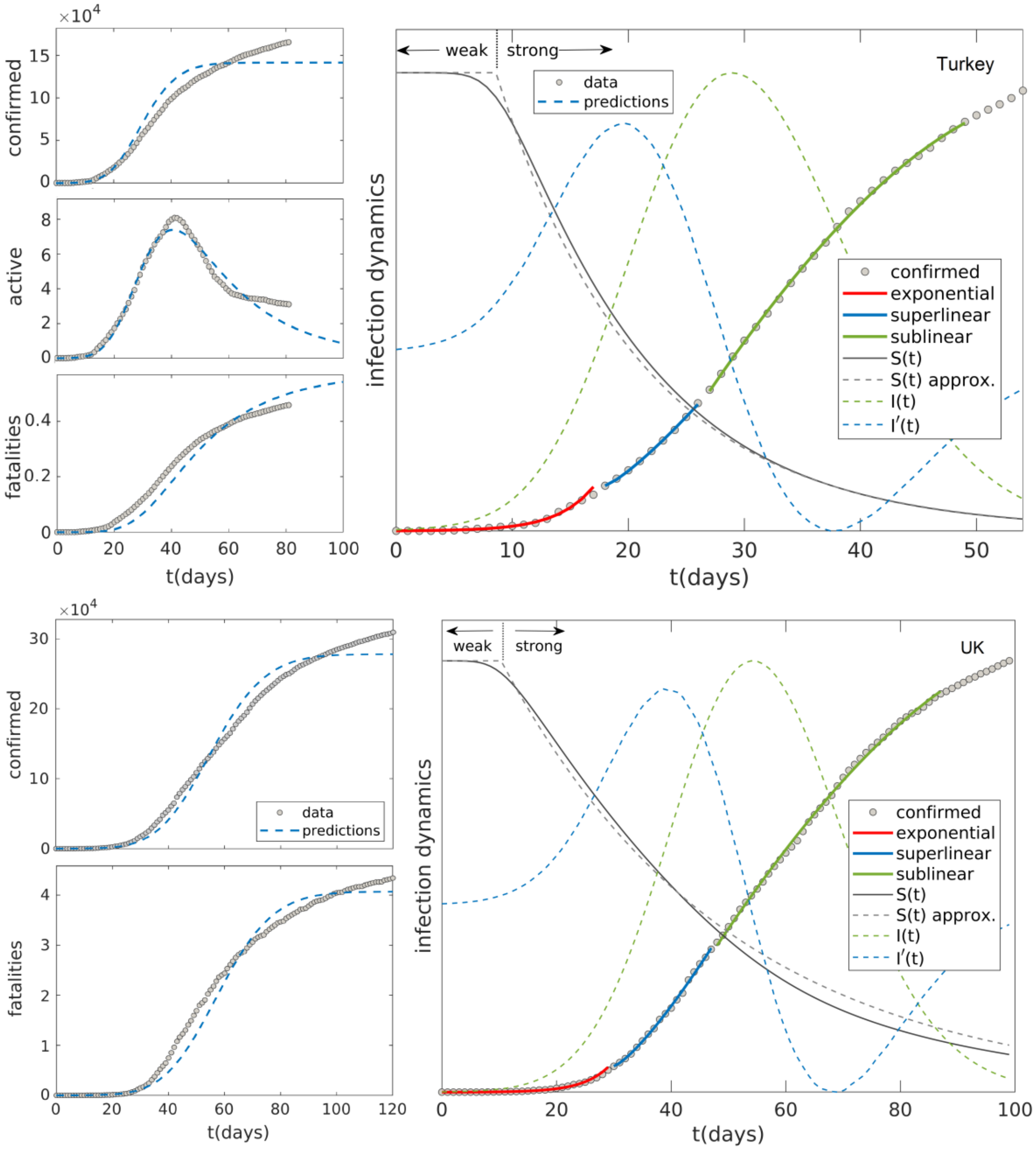
×10^4
confirmed
active
fatalities
t(days)
weak
strong
data
predictions
Turkey
infection dynamics
confirmed
exponential
superlinear
sublinear
S(t)
S(t) approx.
I(t)
I′(t)
t(days)
×10^4
confirmed
data
predictions
fatalities
t(days)
weak
strong
UK
infection dynamics
confirmed
exponential
superlinear
sublinear
S(t)
S(t) approx.
I(t)
I′(t)
t(days)

Country	α range	α	IFR (%)	CFR (%)	AR (%)	Δt (days)	v	R_0
AND	0.03 → 0.33	0.3 ± 0.1	0.6 ± 0.2	8.4 ± 0.8	13 ± 3	5 ± 1	1.4 ± 0.6	2.5 ± 0.3
AT	0 → 0.44	0.4 ± 0.1	0.7 ± 0.3	4.3 ± 0.5	1 ± 1	6 ± 2	1.4 ± 0.2	3.7 ± 0.6
CH	0.02 → 0.17	0.15 ± 0.05	0.2 ± 0.3	7.0 ± 0.7	12 ± 5	4 ± 2	1.27 ± 0.09	3.3 ± 0.4
CRO	0.02 → 0.11	0.10 ± 0.03	0.4 ± 0.3	5.0 ± 0.5	0.8 ± 0.7	7 ± 3	1.1 ± 0.1	3.5 ± 0.6
CU	0.02 → 0.14	0.07 ± 0.03	0.1 ± 0.1	5.4 ± 0.6	1.4 ± 0.6	8 ± 2	1.3 ± 0.1	2.4 ± 0.9
CZ	0.02 → 0.16	0.10 ± 0.02	0.2 ± 0.3	5.8 ± 0.8	2 ± 4	8 ± 1	1.5 ± 0.2	4.0 ± 0.8
DE	0.02 → 0.17	0.07 ± 0.02	0.3 ± 0.2	5.1 ± 0.6	4 ± 3	8 ± 1	1.5 ± 0.1	3.7 ± 0.7
ESP	0.01 → 0.07	0.06 ± 0.01	0.4 ± 0.1	10 ± 1	16 ± 7	13 ± 3	1.47 ± 0.06	4.6 ± 0.4
FR	0.02 → 0.14	0.07 ± 0.02	0.4 ± 0.5	20 ± 2	12 ± 8	14 ± 3	1.51 ± 0.08	3.6 ± 0.6
IL	0 → 0.16	0.09 ± 0.02	0.13 ± 0.07	2.2 ± 0.2	3 ± 2	8 ± 2	1.3 ± 0.1	3.8 ± 0.9
IT	0.01 → 0.033	0.033 ± 0.005	0.7 ± 0.7	15 ± 2	8 ± 9	10 ± 3	1.29 ± 0.08	3.2 ± 0.5
NZ	0.04 → 1.	0.30 ± 0.07	0.3 ± 0.1	1.5 ± 0.1	0.2 ± 0.1	5 ± 1	1 ± 1	5 ± 2
SRB	0.02 → 0.11	0.04 ± 0.01	0.08 ± 0.05	2.3 ± 0.2	5 ± 4	12 ± 3	1.23 ± 0.07	2.9 ± 0.3
TR	0.01 → 0.12	0.08 ± 0.01	0.4 ± 0.1	4.1 ± 0.5	2 ± 1	9 ± 3	1.23 ± 0.05	4 ± 2
UK	0.01 → 0.03	0.025 ± 0.005	0.7 ± 0.2	15 ± 2	9 ± 5	17 ± 3	1.27 ± 0.04	3.1 ± 0.2

Table S1: Notations in the table follow those in Figures 1-3.
